# Understanding the Effect of IM-5 Zeolite Treated with Hexafluorosilicic Acid for the Methanol Alkylation of Pseudocumene

**DOI:** 10.3390/ma18102252

**Published:** 2025-05-13

**Authors:** Shumin Hao, Yongrui Wang, Enhui Xing, Xuhong Mu

**Affiliations:** State Key Laboratory of Petroleum Molecular & Process Engineering, SINOPEC Research Institute of Petroleum Processing Co., Ltd., 18 Xueyuan Road, Beijing 100083, China; haoshumin.ripp@sinopec.com (S.H.); xingeh.ripp@sinopec.com (E.X.)

**Keywords:** IM-5 zeolite, hexafluorosilicic acid, dealumination, silication, pseudocumene, alkylation

## Abstract

A study systematically investigating the structural modifications and catalytic performance of IM-5 zeolite treated with hexafluorosilicic acid in pseudocumene alkylation with methanol was carried out. Characterization techniques revealed significant alterations in crystal structure, morphology, textural properties, coordination environment, and acidity induced by the modifications. Catalytic evaluations demonstrated altered pseudocumene conversion, durene selectivity, and products distribution for optimized samples, with IM-5-0.01 (treated with 0.01 M modifier) showing superior activity stability. The improved performance was attributed to two key factors: a stable framework with high-density medium-strength Brønsted acid sites facilitating complete alkylation and expanded mesoporous volume promoting efficient product diffusion to mitigate deactivation. Conversely, reduced durene selectivity in modified samples stem from intensified isomerization reactions driven by increased external surface area, resulting in higher C_9_ product fractions. In contrast, the parent IM-5 zeolite exhibited rapid deactivation, with durene selectivity peaking at 40 h before declining. Mechanistic insights revealed dynamic processes including dealumination, defect formation, silicon repair, and aluminum redistribution during treatment, providing a theoretical foundation for rational catalyst design in alkylation reactions.

## 1. Introduction

The alkylation of pseudocumene (1,2,4-trimethylbenzene) with methanol served as a foundational pathway for synthesizing durene (1,2,4,5-tetramethylbenzene), a pivotal precursor in high-performance polyimide production [1,2]. While recent advancements explored alternative methodologies—such as syngas conversion [3,4,5], syngas [6], or CO_2_ hydrogenation coupled with alkylation [7]—these approaches inherently depended on bifunctional catalysts to generate methylating agents (e.g., methanol) prior to subsequent alkylation with trimethylbenzenes [8]. Consequently, the classical 1,2,4-TMB/methanol alkylation retained critical relevance for mechanistic exploration and process optimization.

In the 1980s, studies on diverse catalytic systems, including Ag-modified amorphous SiO_2_-Al_2_O_3_ [9], aluminum phosphates [10], montmorillonite catalysts pillared by aluminium hydroxyl complexes [11] and zeolites [12,13,14], revealed the interplay between catalyst structure and durene selectivity. Among these, HZSM-5 zeolites demonstrated molecular sieving capabilities by aligning pore dimensions with the molecular sizes of trimethylbenzene and tetramethylbenzene isomers. Surface passivation using 2,6-dimethylquinoline and acid site modulation via SiCl_4_ treatment effectively suppressed external acid activity, curbing secondary reactions such as isomerization and disproportionation [14]. Nevertheless, HZSM-5’s moderate acid strength and restricted pore aperture (<5.5 Å) limited conversion efficiency, favoring undesired side products like xylenes through disproportionation pathways [15]. These limitations underscored the necessity for shape-selective zeolites with tailored acidity and pore architectures.

IM-5 zeolite (IMF code), first synthesized by Benazzi et al. in 1998 [16], featured a unique 2D/3D pore system comprising intersecting 10-membered ring channels (0.48–0.55 nm) and a 2.5 nm three-dimensional network [17]. Its enlarged pore dimensions compared to MFI-type frameworks (e.g., ZSM-5) enhanced molecular diffusion while maintaining shape selectivity. Notably, IM-5 exhibited superior hydrothermal stability, higher strong acid site density, and improved active center accessibility [18,19,20]. However, the literature studying IM-5 zeolite still used the classic Benazzi method for synthesis currently [21,22,23], which can be attributed that its synthesis remained constrained to narrow SiO_2_/Al_2_O_3_ (30–120) and NaOH/SiO_2_ ratios under conventional hydrothermal conditions [24], necessitating post-synthetic modifications to optimize catalytic performance.

Recent studies highlighted the role of controlled dealumination in tuning IM-5’s acidity and pore structure. Niu et al. [25] demonstrated that nitric acid treatment achieved progressive dealumination, enhancing framework openness while preserving structural integrity. Catalytic cracking evaluations revealed improved activity at SiO_2_/Al_2_O_3_ ratios of 30–120. Conversely, Zhai [26] and Zhang et al. [27] reported that hexafluorosilicic acid treatment optimizes IM-5 for gas-phase alkylation and methanol-to-propylene reactions at SiO_2_/Al_2_O_3_ ratios of 110 and 174, respectively, though these conditions prove suboptimal for 1,2,4-TMB/methanol alkylation. Tao et al. [28,29] systematically modulated Brønsted acid site density in HZSM-5 zeolite via sequential treatment with CF_2_Cl_2_ and SiCl_4_. Controlled experiments revealed that the alkylation of pseudocumene with methanol was preferentially catalyzed by moderate-strength Brønsted acid sites, which optimized the balance between methyl group activation and desorption kinetics.

In this paper, the parent IM-5 zeolite was synthesized via a modified hydrothermal crystallization protocol, followed by systematic modulation of its acidic properties through controlled hexafluorosilicic acid (H_2_SiF_6_) treatment. A comprehensive suite of characterization techniques was employed to interrogate framework integrity, elemental distribution, textural evolution, and acid site density. These analyses aimed to optimize the catalyst for the alkylation of pseudocumene with methanol.

Catalytic performance was evaluated under fixed-bed reactor conditions (360 °C, 0.8 MPa, weight hourly space velocity [WHSV] = 1.55 h^−1^), revealing a structure-activity relationship governed by dynamic evolution involving framework dealumination, mesopore defect formation, silicon reinsertion, and aluminum redistribution during post-synthetic modification. The IM-5-0.01 variant, subjected to concentration-dependent H_2_SiF_6_ etching (0.01 M), exhibited exceptional stability with higher pseudocumene conversion, attributed to its appropriate amounts of middle strong Brønsted acid and a regular and open framework structure.

## 2. Materials and Methods

### 2.1. Materials

The raw materials used in the experiments can be found in the Supplementary Materials.

### 2.2. Synthesis and Modification of IM-5 Zeolites

The parent IM-5 zeolite in this study was synthesized via an optimized two-stage hydrothermal crystallization process, which significantly reduced crystallization duration and minimizes consumption of organic templates, alkaline sources, and water compared to conventional methods. The synthesis protocol employed MPPBr_2_ as the structure-directing agent (SDA) in a PTFE-lined stainless steel autoclave under dynamic conditions.

Aluminum hydroxide and sodium hydroxide were dissolved in deionized water at 80 °C. After cooling to ambient temperature, the density of the solution was measured to calculate aluminum and sodium concentrations. This low-concentration sodium metaaluminate solution served as a partial alkali and aluminum source.

The cooled sodium metaaluminate solution was transferred to the PTFE liner, followed by sequential additions of sodium hydroxide (to achieve the synthesis ratios), deionized water, crude silica gel, and MPPBr_2_. The resulting gel had a molar composition of SiO_2_: 0.025 Al_2_O_3_: 0.185 Na_2_O: 0.14 R: 14 H_2_O.

The homogenized gel was subjected to two-stage crystallization in the autoclave (Parr) under rotational agitation (180 rpm):

- Primary crystallization: 48 h at 140 °C;
- Secondary crystallization: 96 h at 175 °C.

The crystallized product was cooled rapidly, solid-liquid separation via vacuum filtration. After overnight drying at 120 °C, the as-synthesized zeolite was calcined at 550 °C for 5 h under static air to remove organic templates.

The calcined IM-5 zeolite underwent dealumination and silication treatment by mixing with hexafluorosilicic acid solutions (0.005–0.03 M) at a solid-to-liquid ratio of 7.5:1 (*w*/*w*). The slurry was dynamically processed in a PTFE reactor at 80 °C (300 rpm, 1 h), followed by neutralization via repeated deionized water washing and then drying at 120 °C.

All the samples underwent triple ammonium exchange cycles (3 h per cycle) using ammonium chloride (1:1 mass ratio) in 10-fold mass excess of deionized water at 80 °C. The resultant ammonium-form zeolite was converted to its protonic form by calcination at 550 °C for 5 h under static air.

The samples obtained in this way were denoted as IM-5-x, where ‘x’ represented the concentration (M) of the modifying agent, hexafluorosilicic acid.

### 2.3. The Charaterization of Samples

The powder X-ray diffraction (XRD) patterns of the samples were recorded on a D/MAX-IIIA X-ray diffractometer (Rigaku Corporation, Tokyo, Japan) using monochromatized Cu Kα radiation (35 kV, 35 mA). The samples were scanned from 5° to 50° at intervals of 0.013°, with a scanning duration of 30 s for each angle. The bulk chemical composition of the samples was characterized by X-ray fluorescence (XRF) measurement (Rigaku ZSX100E, Rigaku Corporation, Tokyo, Japan) running at 40 kV. A FEI Quanta 200F electron microscope (FEI Company, Hillsboro, OR, USA) operating at 20 kV was used to record the scanning electron microscopy (SEM) images. All NMR experiments were carried out on a Varian IN0VA 300 spectrometer (Vrian Company, Palo Alto, CA, USA). The resonance frequencies for ^29^Si, ^1^H, and ^27^Al were 119.24, 600.23, and 156.4 MHz, respectively. The chemical shifts of ^29^Si, ^1^H, and ^27^Al were referenced to kaolin, adamantane, and 1 M Al(NO_3_) _3_ solution, respectively. Quantitative tests for ^27^Al MAS NMR were performed using a 0.63 (π/12) pulse with a 2 s recycle delay and 1024 scans. Before the ^1^H MAS NMR experiment, the IM-5 samples were dried under vacuum at 420 °C for 20 h. Then, the IM-5 samples were loaded into a 4 mm NMR rotor under a N_2_ atmosphere in the glove box. A spinning rate of 12 kHz, 32 scans, a recycle delay of 10 s, and a π/2 pulse length of 2.70 μs were used for ^1^H MAS NMR. The hydroxyl infrared spectroscopy (OH-IR) of the samples were collected by an FTIR spectrometer (BIO-RAD FTS 3000, Bio-Rad Laboratories Inc., Hercules, CA, USA) in the wavenumber range of 4000–3000 cm^−1^ at 400 °C and under vacuum (pressure < 10^−5^ Pa). Before tests, the samples were pressed into thin wafers and activated at 550 °C for 1 h under vacuum (pressure < 10^−5^ Pa). The temperature-programmed desorption curves of ammonia (NH_3_-TPD) were recorded on an AutoChem II 2920 chemisorption analyzer (Micromeritics Instrument Corporation, Norcross, GA, USA), where the desorption temperature was 600 °C. For the detailed schematic diagram of the NH_3_-TPD, please refer to Figure S1. Pyridine was used as the probe molecule to determine the concentration and distribution of acid sites by infrared spectroscopy. The spectra were also measured with a spectrometer (BIO-RAD FTS 3000) in the wavenumber range of 1700–1300 cm^−1^. Like the OH-IR, the samples also underwent pre-treatment. After the samples cooled to room temperature, the spectra were recorded. Pyridine was adsorbed for 0.5 h at room temperature. The strength of the acid sites was probed by increasing the temperature to 190 °C, 310 °C, and 410 °C. Thermal analysis experiments were carried out using a Netzsch STA 409CD analyzer (NETZSCH-Gerätebau GmbH, Bavaria, Bundesrepublik Deutschland) in a flow of air or argon (100 mL/min) at 10 °C/min from 30 to 1200 °C.

### 2.4. Catalyst Testing

The catalytic tests were carried out in a fixed-bed down-flow tubular reactor system (ShenYang ShiBoDa Instrument Co., Ltd., Shenyang, China). The reaction tube was 50 cm in length and had an inner diameter of 8 mm. Two grams of proton—type molecular sieves were tableted, respectively, and screened to an appropriate particle size range (0.425–0.85 mm) before being loaded into the reactor. An HPLC pump (LabAlliance, State College, PA, USA) was used to introduce the liquid feed into the reactor, while a mass flow controller (Brooks Instrument, Hatfield, PA, USA) was utilized to measure the gas flow rate. The liquid feed was pre-mixed with nitrogen prior to entering the reactor, and the system pressure inside the reactor was controlled by a back-pressure regulator (TESCOM Corporation, Elk River, MN, USA).

After the reaction, the products were separated by a three-way joint. Part of the products entered a six-way valve for online gas chromatography analysis, and the other part flowed into a water-cooled liquid storage tank. The gas chromatograph employed was a VARIAN GC 450 (Vrian Company, Visalia, CA, USA) equipped with a flame ionization detector (FID). The chromatographic column was an HP-INNOWAX (Agilent Technologies, Santa Clara, CA, USA), with nitrogen serving as the carrier gas.

The catalyst activation process was as follows: Under the condition of nitrogen flow, the temperature of the reactor was gradually increased (1.5 °C/min) to 500 °C and maintained for 8 h. After that, the temperature was decreased to the reaction temperature. The reaction conditions for the alkylation of pseudocumene with methanol were as follows: the temperature was 350 °C, the pressure was 0.8 MPa, the molar ratio of pseudocumene to methanol was 2:1, and the weight hourly space velocity (WHSV) was 0.5 h^−1^.

The conversion rates of pseudocumene and the selectivity of each product were calculated using the following formulas:

(1) $$X_{TMB}=\frac{{{(m}_{TMB})}_{feed}-{{(m}_{TMB})}_{product}}{{{(m}_{TMB})}_{feed}}\times 100$$

(2) $$S_{PRODCUT}=\frac{m_{PRODUCT}}{m_{TOTAL}-{{(m}_{ME})}_{product}-{{(m}_{TMB})}_{product}}\times 100$$

where the ${m\;}_{TMB}$ and $m_{PRODUCT}$ were the weight of pseudocumene and products, respectively.

## 3. Results

### 3.1. The Phase, Morphology and Textural Properties of IM-5 Zeolites

#### 3.1.1. Phase

**Figure 1:**
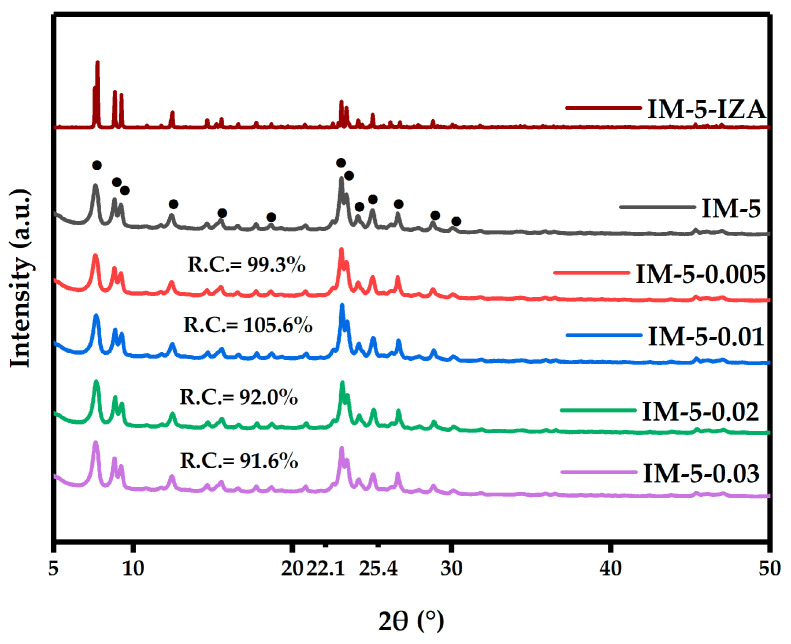
XRD patterns of the samples. The black dots represent the positions of the characteristic XRD diffraction peaks of the IMF topological structure. The R.C. and its accompanying numerical values denote the relative crystallinity (expressed in percentage terms), defined as the ratio of the integrated intensity sum of the most intense diffraction peaks within the 2θ angular range of 22° to 27° to that of the parent zeolite. IM-5-IZA represents the XPD curve of the standard IMF-type zeolite from the Structure Commission of the International Zeolite Association (SC-IZA).

The diffraction peaks observed at 2θ = 7.65°, 8.87°, 9.28°, 12.40°, 15.46°, 18.67°, 23.11°, 23.42°, 24.18°, 25.05°, 26.63°, 28.88° and 31.10° of all the IM-5 samples indicated a typical IMF topological structure (Figure 1) [30,31]. No other phases were detected in the patterns, indicating that pure IM-5 zeolites had been obtained.

Interestingly, the relative crystallinity of the samples showed a trend of “decrease-increase-decrease”, which may be related to the dynamic equilibrium of dealumination and silicon supplementation of IM-5 zeolite by hexafluorosilicic acid [26,32].

#### 3.1.2. Morphology

**Figure 2:**
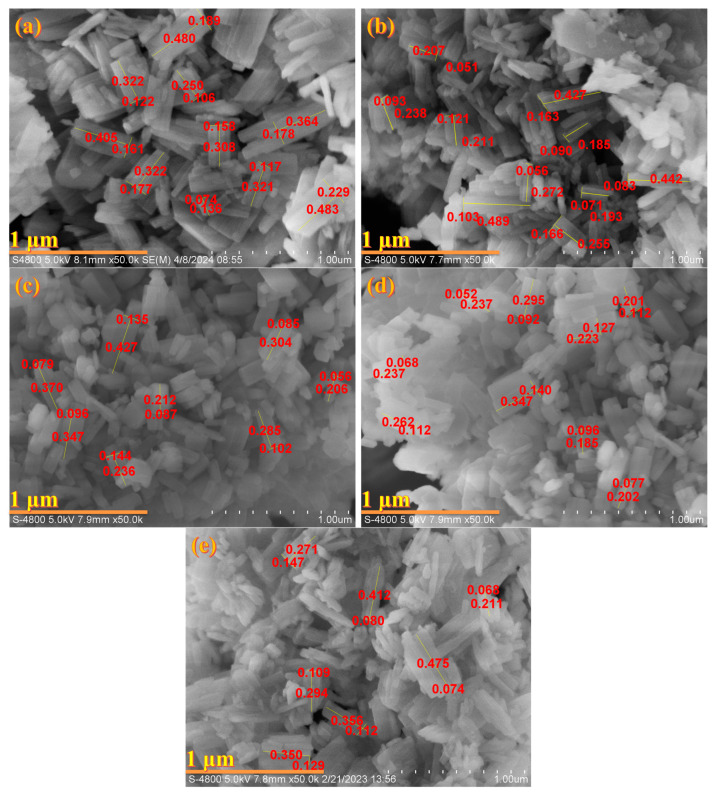
SEM images of the samples: (**a**) IM-5; (**b**) IM-5-0.005; (**c**) IM-5-0.01; (**d**) IM-5-0.02; (**e**) IM-5-0.03. Red and green numbers, respectively, indicate the grain sizes of the zeolite crystals and graphic scales, µm.

As shown in Figure 2a, IM-5 zeolite was observed as flat plates with wrinkled surfaces and dense structure, whose average grain size was 195 nm in length and 76 nm in width. However, after being treated with hexafluorosilicic acid, with the increase in the concentration of the reagent, the surface of the zeolite crystals gradually became smooth, and the grain size of the crystals gradually decreased (Figure 2b–e). Typically, the IM-5-0.03 sample obtained by treating with a 0.03 mol/L hexafluorosilicic acid solution, most of the grains become small and irregular, and only a minimal quantity of the crystals adhered to the inherent plate morphology (Figure 2e). The structural defects induced by preferential dealumination diminished crystal integrity by disrupting lattice periodicity. Silication processes preferentially localized at high-energy crystal edges and defect sites, leading to progressive erosion of angular features and concentric shrinkage of crystallites from the surface inward, as previously reported [27]. Microporous and mesoporous domains formed during dealumination introduce heterogeneous stressed distributions within the crystalline framework. Under mechanical agitation or ultrasonic treatment, these defect-prone regions acted as initiation sites for fragmentation, yielding smaller particles through stress-induced breakage. This “self-abrasive” mechanism was particularly pronounced in traditional fluoride aqueous treatments, where F^−^ ions enhanced structural vulnerability via accelerated dissolution at defective interfaces, exacerbating crystallite disintegration [33].

Compared with the non-monotonic trend (“decrease—increase—decrease”) of the relative crystallinity in the XRD pattern (Figure 1) with the concentration of the modifier, the SEM images in Figure 2 showed that the crystals were gradually broken. This phenomenon may be rationalized by the fact that there was no inevitable connection between relative crystallinity and the integrity of the crystal morphology, but there may be a conditional correlation, especially for the post-treatment modification process of the zeolite rather than the hydrothermal crystallization synthesis process. The relative crystallinity was characterized by XRD, which reflected the long-range order of the crystal structure (that is, the degree of integrity of the lattice periodicity) [34]. High crystallinity meant fewer lattice defects, high and sharp diffraction peak intensities, but it only represented the orderliness of the internal structure of the crystal, rather than the macroscopic integrity of the particles. High-crystallinity materials may lead to particle fragmentation due to post-treatment (such as dealumination), but the crystallinity can still be maintained or even improved (only the removal of non-framework components, and the remaining structure is ordered) [35].

#### 3.1.3. Textural Properties

**Figure 3:**
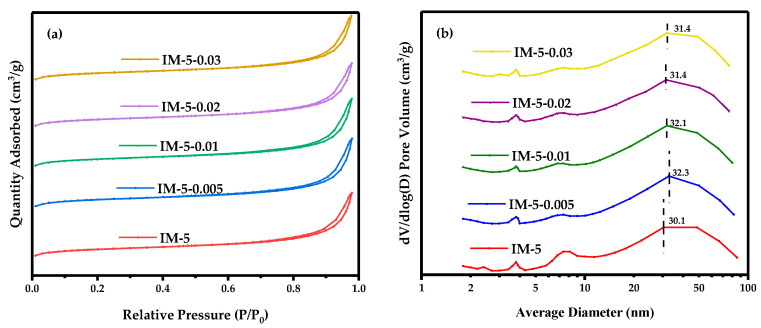
N_2_ adsorption-desorption isotherms (**a**) and BJH desorption pore size differential distribution curve (**b**) of the samples. Black numbers and their dashed lines in (**b**) indicate the most accessible pore size of the samples, nm.

As shown in Figure 3a, all the samples had the combined characteristics of type I and type IV isothermal adsorption curves with one closed hysteresis curves with mesoporous characteristics in the range of higher relative pressure of 0.45~0.99, indicating that the mesopores were mainly intergranular pores formed by the accumulation of nano-zeolite crystals, which was further confirmed by the pore size differential distribution curve obtained by the BJH method (Figure 3b).

The pore size differential distribution curve showed that the most accessible pore size of all the modified IM-5 zeolites were larger than the parent IM-5 zeolite, nevertheless which reduced more and more in size, with the increase in the concentration of the modifier (Figure 3b).

Table 1 listed that as the concentration of the modification reagent increased, the total specific surface area and the microporous area of the samples gradually decreased. Similarly, the microporous volume also decreased. However, the external surface area and the mesoporous volume first increased and then decreased, which corresponds to the experimental results of the most accessible pore diameter in BJH desorption.

Hydrofluorosilicic acid dissociated in solution to form H^+^ and SiF_6_^2−^. The acidic H^+^ environment enabled SiF_6_^2−^/F^−^ to coordinate with framework Al species, extracting aluminum as AlF_6_^3−^ complex ions. This preferential etching at weak framework sites (e.g., micropore walls) caused micropore collapse/merging, reducing micropore area/volume [36]. Collapsed micropores expanded into mesopores, with interconnected new pores increasing mesopore and total pore volumes [37]. External surface area, derived from particle surfaces rather than micropore interiors, may rise as etching exposes internal pores or creates new surfaces via fragmentation [38]. At concentrations > 0.01 M, hydrolyzed (Si(OH)_4_ from (NH_4_)_2_SiF_6_ filled dealumination defects, preferentially depositing on particle surfaces/pores to form amorphous silicon layers, increasing roughness [36].

### 3.2. The Coordination Structure of IM-5 Zeolites

Next, the coordination relationship of Si, Al, and H species during the modification process was studied by MAS NMR, and the hydroxyl distribution of zeolites was also investigated by OH-IR.

#### 3.2.1. ^27^ Al MAS NMR

**Figure 4:**
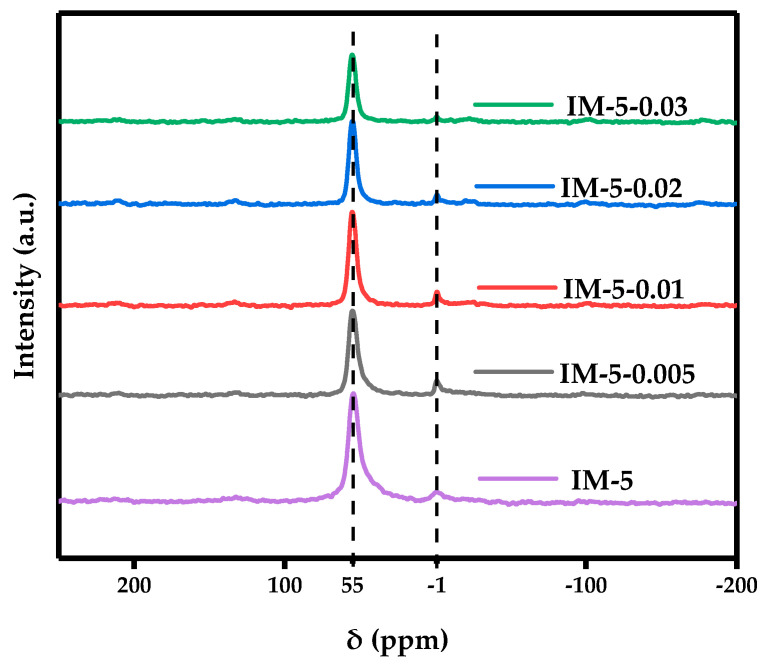
^27^ Al MAS NMR spectrum of the samples. The dashed lines indicate the chemical shifts of the resonance signal peaks.

Figure 4 showed the coordination situation of Al species in all samples. According to the literature [22], the resonance peaks at around 55 ppm and −1 ppm in chemical shift were attributed to framework Al species in tetra-coordinated and extra-framework Al species in hexa-coordinated, respectively. The resonance peaks representing extra-framework Al species in hexa-coordinated first increased and then decreased in intensity until they almost disappeared. However, the resonance peak intensity corresponding to framework Al species in tetra-coordinated exhibited a non-monotonic fluctuation—initial attenuation was followed by transient enhancement before eventual diminishment. This complex behavior suggests a concentration-dependent competition between two processes: (i) selective removal of framework Al species in tetra-coordinated via dealumination at lower modifier concentrations (0.005 M), and (ii) potential structural reorganization facilitating Al reincorporation into the framework at intermediate concentrations (0.01~0.02 M). Ultimately, (iii) under higher modifier concentration conditions (0.03 M), Al species in both coordination forms were removed. This indicated that the dealumination effect of hexafluorosilicic acid on the IM-5 zeolite targeted Al species in the two coordination forms.

#### 3.2.2. ^29^ Si MAS NMR

**Figure 5:**
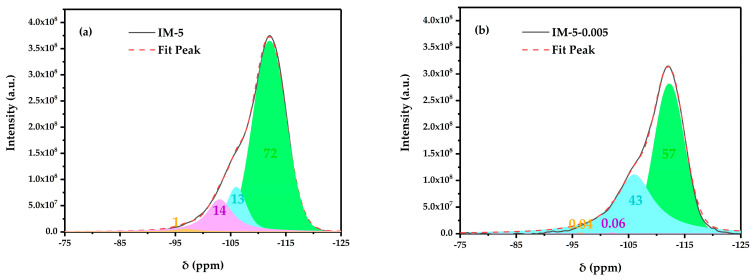

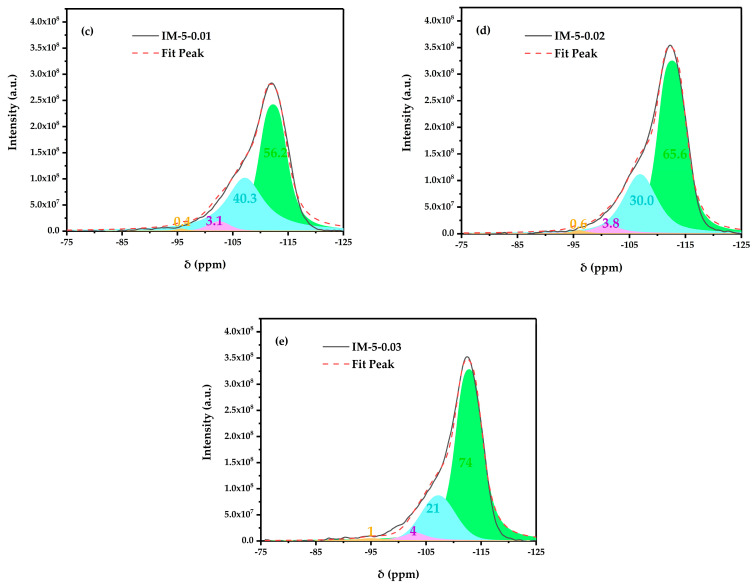
^29^ Si MAS NMR spectrum of the samples: (**a**) IM-5; (**b**) IM-5-0.005; (**c**) IM-5-0.01; (**d**) IM-5-0.02; (**e**) IM-5-0.03 and their deconvolution into Gaussian peaks. The black solid line is the experimental curve. The red dotted line is the cumulative curve of Gaussian peaks. Numbers indicate the percentage corresponding to the resonance peaks, %.

The resonance peaks around −113, −106, −103, and −96 ppm were regarded as Si(0Al), Si(1Al), Si(2Al), and Si(3Al) in IM-5 zeolite, respectively [25]. As clearly illustrated in Figure 5, the resonance signal intensity of Si(0Al) displayed a distinct two-stage evolution: an initial decline followed by a subsequent recovery, whereas the Si(1Al) signal demonstrated an inverse trend. The 0.01 mol/L modifier concentration served as a critical threshold in this modification process, marking the transition from dominance of dealumination to silication. This observation highlighted the dynamic structural rearrangement and synergistic redistribution of Al/Si species within the zeolite framework during the modification.

Owing to the inherently low Si^4+^ concentration in hexafluorosilicic acid aqueous solution, preferential dealumination prevailed during the low-concentration modifier treatment, generating framework defects and inducing structural amorphization. In contrast, at a moderate concentration (0.01 mol/L), the efficiency of silication was improved. Progressive Si incorporation reduced the framework Al content (manifested by increasing Si/Al ratio, especially that of framework), leading to increased Al-Al atomic spacing. The resulting decrease in Al density directly attenuated the Si(1Al) signal intensity. Concurrently, Si deposition effectively repaired the framework discontinuities caused by dealumination, restoring long-range order and increasing the proportion of Si(0Al). At excessively high modifier concentrations (≥0.02 mol/L), dealumination kinetics outpaced silication rates, precipitating a pronounced decline in relative crystallinity. This mechanism accounted for the characteristic “decline-recovery-decline” pattern observed in XRD-measured relative crystallinity.

#### 3.2.3. ^1^ H MAS NMR

**Figure 6:**
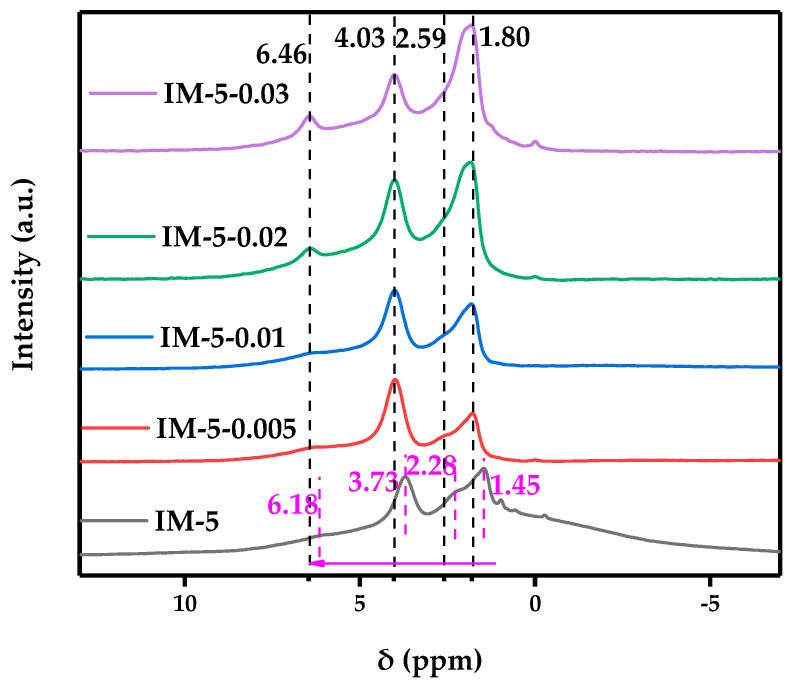
^1^ H MAS NMR spectrum of the samples. The numbers (whose units are nm) and dashed lines represent the chemical shifts of the resonance signal peaks, among which the black font represents the modified samples, and the magenta font refers to the parent IM-5 sample. The magenta arrow indicates the shifting direction of the resonance signal peak of the modified sample.

As shown in Figure 6, the resonance signal peaks of the modified sample were mainly concentrated at chemical shifts of approximately 1.80, 2.59, and 4.03 ppm, which can be attributed to the protons of silanols, the protons of EF-Al, and Si–OH–Al (BAS), respectively. With the increase in the concentration of the modifier, the signal intensity of the protons of silanols exhibited an initial attenuation followed by subsequent augmentation, the signal intensity of the protons of EF-Al demonstrated a marginal initial rise before abruptly subsiding, whereas the signal intensity of Si–OH–Al (BAS) showed a steep surge prior to a precipitous decline.

In addition, the modified sample exhibited a resonance signal peak at a chemical shift of 6.46 ppm, especially for the samples with a relatively high concentration of the modifier. Interestingly, each resonance signal peak of the modified sample shifted towards a higher δ value, with a displacement of approximately 0.3 to 0.4 ppm. Both phenomena can be ascribed to Brønsted acid sites disrupted by hydrogen bonding interactions, which exhibited reduced stability [39]. While silication enhanced framework stability, it concurrently induces heterogeneous distribution of aluminum species within the zeolite lattice. Such compositional heterogeneity led to insufficient Si atoms in the vicinity of certain Al atoms, impeding the establishment of stable chemical linkages. Consequently, these under-coordinated Al sites became predisposed to hydrogen bond formation with water molecules or other oxygen-containing moieties. Moreover, an increase in the intensity of hydrogen-bonding interactions will significantly enhance the deshielding effect of hydroxyl protons, causing their chemical shifts to move towards the downfield (higher δ value) [39]. This indicated that under the action of a high-concentration modifier, the redistribution of aluminum species occurred.

#### 3.2.4. OH-IR

**Figure 7:**
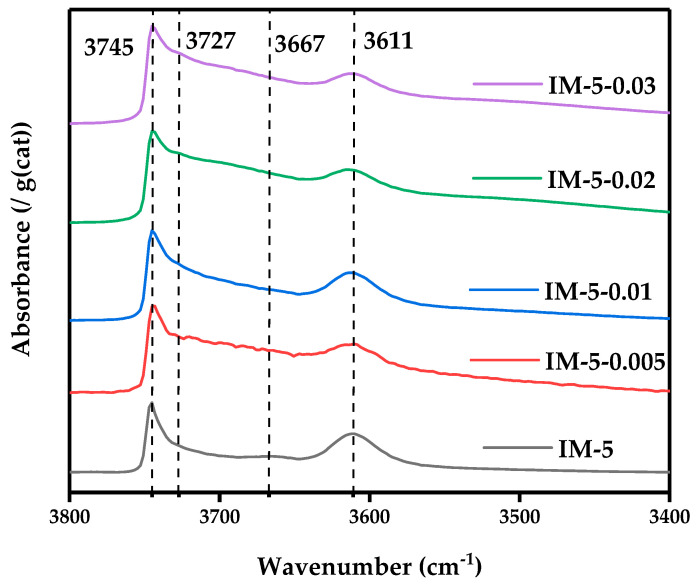
The OH-IR spectrum of the samples. The numbers and dashed lines represent the wavenumbers of the absorption peaks.

Figure 7 showed all the samples had characteristic absorption peaks at wavenumbers of 3745, 3666, and 3610 cm^−1^, representing Si-OH groups on external surfaces, Al-OH extra-framework, and Si-OH-Al, respectively [25].

As the concentration of the modifier increased, the absorption peak of the Si-OH groups on external surfaces was first on the downside and then rose, the absorption peak of the Al-OH extra-framework almost disappeared, and the absorption peak of Si-OH-Al showed a sawtooth trend that first rose, then fell, and finally rose again. This aligned with the findings of NMR characterization (Figure 4 and Figure 6).

In addition, the modified sample showed a characteristic absorption peak at a wavenumber of 3727 cm^−1^, and it became stronger as the concentration of the modifier increased. After the tetracoordinate framework Al species (existing as AlO_4_ tetrahedrons) was removed, structural vacancies would be left behind. If the silication process lagged, these vacancies cannot be filled in a timely manner by silicon species. The surrounding silicon atoms could form silanol groups (Si-OH) to compensate for the local charge or structural imbalance, forming “silanol nests” [25]. This validated the earlier hypothesis that silication lagged behind aluminum removal when samples were treated with high concentrations of the modifier. Silanol nests were essentially local structural defects in the framework. Although the bond energy of the Si-O bond was relatively high (more stable than the Al-O bond), an excessive number of unrepaired silanol nests would lead to stress concentration or structural looseness in the framework, creating weak points in terms of thermal stability and hydrothermal stability [40]. At high temperatures, silanol groups may dehydrate to form siloxane bonds (Si-O-Si), but excessive dehydration would cause the framework to shrink or the pore structure to collapse. Under hydrothermal conditions, the hydroxyl groups easily interacted with water molecules, intensifying the dissolution or reconstruction of the framework, and making the material more prone to structural collapse or crystal form transformation under harsh conditions (such as high temperature, high pressure, and acidic environments) [41]. During the process of dealumination and silicon supplementation, it was necessary to control the balance between dealumination and silicification, avoid the excessive formation of silanol nests, and ensure that silicon species can fill the defects in a timely manner, so as to maintain or enhance the stability of the molecular sieve framework. Therefore, a moderate concentration of the modifier was of great importance for the stability of the zeolite framework.

### 3.3. The Acidity of IM-5 Zeolites

The TPD curve was deconvoluted into three peaks, which correspondingly represented weak acid sites with the peak center at T_1_ = 180~200 °C, middle strong acid sites with the peak center at T_2_ = 270~370 °C, and strong acid sites with the peak center at T_3_ = 400~430 °C (Figure S4a–e).

**Figure 8:**
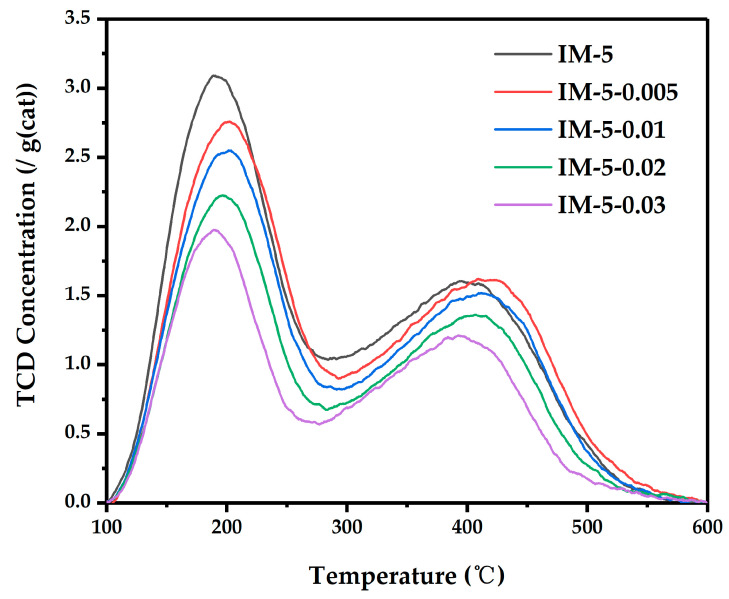
NH_3_-TPD curves of the samples.

As shown in Figure 8 and Table 2, the bulk n(SiO_2_)/n(Al_2_O_3_) of all samples increased with the increasing in the concentration of the modification reagent, which was consistent with what was described in the literature. Interestingly, the Si/Al ratio of parent IM-5 zeolite was lower than that of the precursor gel. This can be ascribed that the hydrothermal crystallization of IM-5 zeolite occurred under highly stringent conditions, characterized by narrow operational windows for raw material stoichiometry, temperature, and crystallization duration. Concurrently, partial incorporation of the silicon source into the silicoaluminate framework was thermodynamically restricted, leading to a reduced Si/Al ratio in the synthesized zeolite. This phenomenon was corroborated by established literature [24] and mirrored in recent studies [21].

The total acid amounts and the amounts of weak acid of all samples also decreased accordingly. This indicated that hexafluorosilicic acid played a role in dealumination and silicon replenishment for the samples. Interestingly, the amounts of medium acid showed a trend of first increasing and then decreasing, while the amounts of strong acid showed the opposite trend, and the sample IM-5-0.01 had the highest amount of medium acid. In addition, the desorption temperatures of the three types of acids all showed a trend of first increasing and then decreasing. The literature had proven that the desorption temperature of TPD was related to the acid strength, and similarly, the sample IM-5-0.01 had the highest desorption temperature.

**Figure 9:**
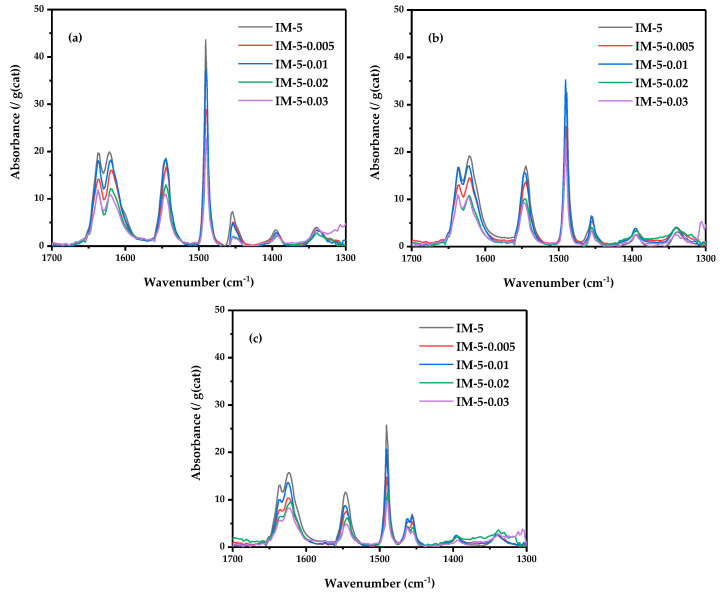
Py-IR curves of the samples at: (**a**) 190 °C; (**b**) 310 °C; (**c**) 410 °C.

Similarly, infrared spectroscopy experiments of pyridine adsorbed at three different desorption temperatures were carried out for three groups, corresponding to the three different acid types classified by the NH_3_-TPD profiles. Consistent with the pattern of TPD, the total acid amounts of the modified samples all decreased. However, the acid amount of IM-5-0.01 almost remained unchanged and was the highest among all the modified samples, whether it was the amount of Brønsted acid or Lewis acid. This may be related to the balance between dealumination by hexafluorosilicic acid and silicon replenishment. Herein, its B/L value at each desorption temperature was also relatively high. (Table 3 and Figure 9). When the modifier concentration was 0.005 M, tetracoordinate framework Al species was preferentially removed, accompanied by an increase in the signal intensity of hexacoordinate extra-framework Al species (as observed via ^27^Al MAS NMR and OH-IR spectroscopy). Consequently, the B/L ratio decreased significantly (from 0.5 to 0.7). At a modifier concentration of 0.01 M, defect repair via silication commences: tetracoordinate framework Al species showed a rebound trend, while the signal intensity of hexacoordinate extra-framework Al species weakened (^27^Al MAS NMR and OH-IR). Due to the dynamic nature of the dealumination-silication process, these changes were less pronounced compared to the parent and IM-5-0.005 samples. Treatment with a higher modifier concentration induced synergistic dealumination and silication, with dealumination dominating: tetracoordinate framework Al species decreased again, and hexacoordinate extra-framework Al species signals nearly disappeared (^27^Al MAS NMR and OH-IR). These results were in a significant increase in the B/L ratio at 190 °C (~1) and a substantial decrease at 410 °C (~1.4). Notably, the B/L ratio of middle strong acids (310 °C) exhibited little change or a slight increase (−0.2 to +0.3), consistent with the increased middle strong acid amount observed in NH_3_-TPD. This was attributed to middle strong acid sites being associated with aluminum species in relatively stable framework coordination environments (e.g., Al^3+^ with middle adjacent silanol groups) or defect sites formed during partial dealumination. In the early dealumination stage, these sites were preserved because the aluminum coordination environment remained largely intact. During silication (0.01 M modifier), introduced silicon species (e.g., silanols or siloxane polymers) partially repaired framework defects, forming new middle strong acid sites—for example, in Si-O-Al structures where reduced aluminum charge density moderated acidity. Zeolitic strong acid sites primarily arises from Brønsted acids (B acids, e.g., Si-OH-Al structures) from tetracoordinate framework Al species and highly active extra-framework aluminum Lewis acids (L acids). During dealumination, strong acid sites were vulnerable to attack by acidic reagents (NH_4_^+^, H^+^, or fluorides) due to the high charge density and low stability around aluminum [42], leading to coordination bond cleavage and aluminum detachment from the framework.

### 3.4. Catalytic Property Test

**Figure 10:**
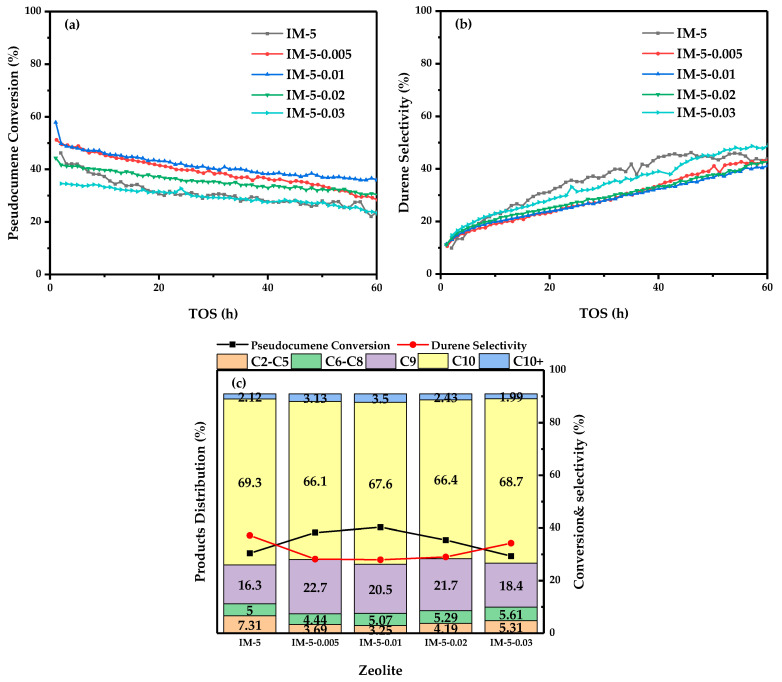
Catalytic performance evaluation of the samples: (**a**) pseudocumene conversion; (**b**) durene selectivity; (**c**) product distribution at 30 h, with numerical values indicating the percentage composition of each product category in %.

A moderate modifier concentration (0.01 M) endows the specimen with the highest pseudocumene conversion efficiency in catalytic performance (Figure 10a). This phenomenon is attributed to two key factors: (1) the stable framework structure combined with a high density of medium-strength Brønsted acid sites, which facilitate complete alkylation reactions; (2) the large mesoporous volume that promotes efficient product diffusion from the pore channels, thereby mitigating catalyst deactivation. This improved stability is manifested by the significant C_10_+ product fraction observed in the distribution profile.

Notably, all modified samples exhibit reduced durene selectivity (Figure 10b). This decline can be primarily ascribed to enhanced pseudocumene isomerization reactions driven by increased external surface area after modification, resulting in a higher proportion of C_9_ products in the distribution. According to the research findings of Mykela DeLuca and David Hibbitts [43], the critical diameters of the reactant pseudocumene and the main product durene were the smallest among the trimethylbenzene and tetramethylbenzene isomers, both being 7.3 Å. Over MFI-type zeolite, the diffusion potential energies of pseudocumene and durene in the straight channels and sinusoidal channels were also the lowest among their respective isomers [43]. The pore size of IM-5 zeolite on the [010] crystal plane was slightly larger, but the pore size along the direction of the X-axis was comparable or even the two-dimensional channel systems on both sides were smaller. Moreover, different from the complete three-dimensional 10-MR channel system of MFI-type zeolites, the IMF-type zeolites had a three-dimensional nanoporous system with a thickness of 2.5 nm, which was formed by three two-dimensional channels in the center and on both sides, and these nanoporous systems were not interconnected [17]. In the research results of H.P. Rŏger and others [44], it has been confirmed that the isomerization of pseudocumene to trimethylbenzenes on ZSM-5 zeolite mainly occurred on the external surface. Therefore, by analogy with MFI-type zeolites, the insignificant change in the pore size after the modification of IMF-type zeolites was not the key factor for the large-scale generation of trimethylbenzenes, and the desorption and diffusion of durene were also not greatly affected by the modification. Considering the increase in the selectivity of C_9_ aromatics for the IM-5-0.01 sample in the product distribution of this paper (Figure 10c) and the increase in the external surface area (Table 1), it was believed that the decrease in the selectivity for durene was due to the large-scale generation of trimethylbenzenes.

In Figure 10c, the product can be classified into non-aromatics (i.e., C_2_–C_5_), benzene, toluene, and xylene (i.e., C_6_–C_8_), trimethylbenzene (C_9_), tetramethylbenzene (C_10_), pentamethylbenzene, and hexamethylbenzene (C_10_+). Figure S5 showed the chromatographic effluent curve of the reaction over the parent IM-5 zeolite under typical conditions. In previous studies [15], it has been found that under the given reaction conditions, regarding the product distribution over the IM-5 zeolite, the non-aromatics, benzene, and toluene mainly originated from the MTH reaction, the xylene come from the paring and disproportionation reactions of pseudocumene, the trimethylbenzene was derived from the isomerization reaction of pseudocumene, the tetramethylbenzene was from the alkylation reaction, and the C_10_+ was from the deep alkylation reaction, which was quite different from the reaction pathway of the ZSM-5 zeolite. Under the conditions of this article, for the samples modified with fluosilicic acid, due to the slight change in the pore size distribution (Figure 3b), the selectivity for tetramethylbenzene has not been significantly improved. However, due to the enhanced activity of the external surface area, the increased mesoporous volume made the active centers more accessible, and the higher density of middle strong acids that promoted alkylation, the product distribution of the isomerization product trimethylbenzene with a larger critical diameter and the deep alkylation products pentamethylbenzene and hexamethylbenzene has been significantly improved. Correspondingly, more methanol participated in the alkylation reaction, the MTH reaction was weakened, and the product distribution of the generated non-aromatics, benzene, and toluene decreased. Since the paring reaction was mainly affected by the reaction temperature [45] and the bimolecular mechanism of the disproportionation reaction was restricted by the pore channels [46], the product distribution of xylene changed little.

In contrast, the parent IM-5 zeolite demonstrates pronounced deactivation behavior. Its durene selectivity reaches a maximum equilibrium value after 40 h of operation, followed by a discernible downward trend.

These results highlight that moderate hexafluorosilicic acid treatment of IM-5 zeolite holds substantial potential for enhancing catalyst activity stability in industrial applications.

The sample IM-5-0.01 exhibited high activity stability and conversion due to its larger mesoporous volume and abundant medium-strength Brønsted acid sites. Notably, the parent IM-5 zeolite showed high initial durene selectivity but reached equilibrium at 40 h (Figure 10b). At high catalyst activity, the reaction predominantly followed the alkylation pathway to durene, with selectivity rapidly reaching equilibrium. As catalyst deactivation occurred, the main reaction rate declined more significantly, while side reactions (e.g., durene isomerization or MTA), which required fewer active sites, gradually dominated—causing selectivity to decrease only after a prolonged post-equilibrium period.

**Figure 11:**
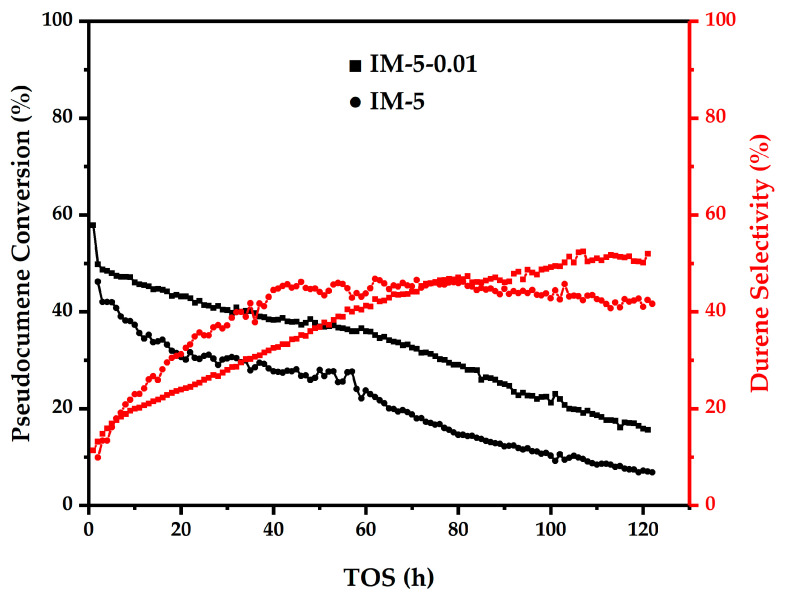
Results of the catalytic evaluation in the 120-h long-period operation of the samples.

To further assess the modifier’s effect on zeolite catalysis, a 120-h long-term alkylation experiment was conducted (Figure 11). Throughout the reaction, IM-5-0.01 maintained a 10% higher pseudocumene conversion than the parent zeolite, with durene selectivity steadily increasing and surpassing the parent after 80 h. The parent zeolite, with abundant strong acid sites, underwent intense early-stage side reactions, leading to rapid coke deposition, deactivation, and steeper conversion decline. In contrast, IM-5-0.01 featured moderate acidity, stable active sites, an open pore system, and reduced diffusion limitations—resulting in lower coke formation rates and consistently higher conversion.

The improved durene selectivity in the two samples stem from dynamic reaction pathway competition and divergent coking behavior:

1. Initial stage: Strong acid sites in the parent zeolite accelerated alkylation but concurrently drove side reactions (e.g., polyalkylation, coking), boosting early durene selectivity. IM-5-0.01’s reduced acidity slowed the main reaction slightly, suppressing side reactions and yielding temporarily lower selectivity.
2. Middle stage: Parent zeolite deactivated rapidly due to coke-covered strong acid sites, reducing main reaction rates and increasing isomerization dominance—lowering durene selectivity. IM-5-0.01’s stable acid sites sustained the main reaction, maintaining durene formation and driving selectivity past the parent.
3. Later stage: While both samples approach equilibrium selectivity, the modified zeolite achieved a higher plateau and stability, attributed to superior diffusion and acid-site resilience.

**Figure 12:**
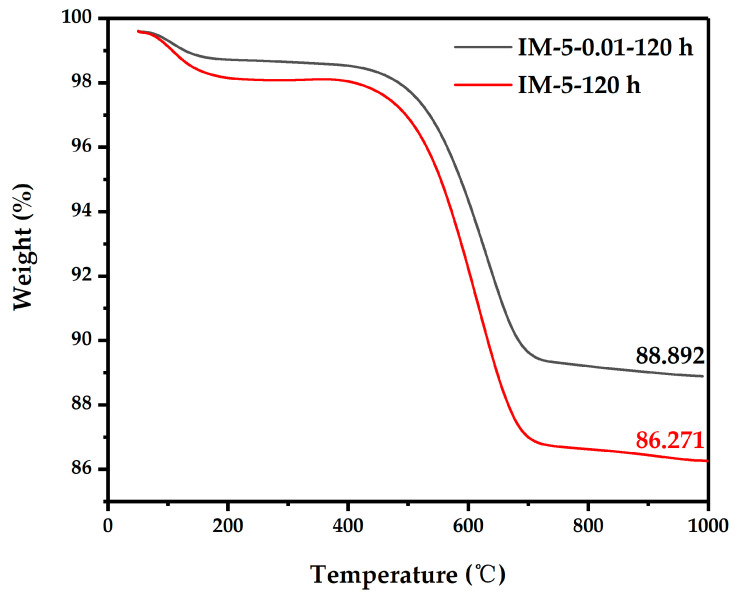
TGA curves of the samples.

Thermogravimetric analysis of spent samples (Figure 12) revealed that the parent zeolite’s strong acids promote aromatic condensation and coke deposition, blocking pores and active sites—leading to higher coke loads. IM-5-0.01’s moderate acidity and unobstructed pores delayed coking, preserving active site accessibility and sustaining high conversion. Even with increased C_10_+ formation (Figure 10c), desorption and diffusion remain facile, minimizing selectivity penalties from coke deposition.

## 4. Discussion

During the modification process, the evolution of Si-Al coordination environments exerted a significant impact on the pore architecture and mass transport properties of zeolites. High Si/Al ratio zeolite samples typically exhibited enlarged mesopore diameters and enhanced external surface areas. This phenomenon can be attributed to the framework regularization induced by Si substitution, which mitigated structural defects and optimized channel connectivity, thereby reducing intracrystalline diffusion resistance.

The coordination structural evolution of modified samples reveals that at low modifier concentrations (0–0.005 M), initial dealumination occurs primarily targeting framework aluminum. This process results in enhanced resonance signals for hexacoordinate extra-framework Al species, decreased Si(0Al) populations, increased Si(1Al) proportions, and persistently strong EF-Al signals alongside Al-OH extra-framework absorption bands. Under moderate concentrations (0.005–0.01 M), silicon replenishment efficiency improves progressively while extra-framework aluminum removal remains dominant. This is characterized by intensified tetracoordinate framework Al resonances, continuous reduction of Si(0Al), and concurrent weakening of EF-Al signals and Al-OH absorption features. At high concentrations (>0.01 M), silicon replenishment fails to keep pace with dealumination dynamics, leading to the formation of silica hydroxyl nests. Concomitant structural changes include attenuated tetracoordinate framework Al resonances, diminished Si(1Al) signals, near-disappearance of EF-Al and Al-OH signatures, and perturbation of Brønsted acid sites due to hydrogen bond interactions.

The IM-5-0.01 catalyst exhibited superior pseudocumene conversion efficiency (58%→40% over 60 h), demonstrating a 10% enhancement compared to the parent IM-5. Notably, the conversion hierarchy (0.01 M > 0.005 M > 0.02 M > 0.03 M ≈ pristine) showed monotonic correlation with middle strong acid sites quantified by NH_3_-TPD, confirming acid-catalyzed alkylation as the rate-determining step.

Importantly, durene selectivity over IM-5-0.01 remained the most suppressed (12%→40%), 10% lower than the parent zeolite. This selectivity hierarchy (pristine > 0.03 M > 0.01 M ≈ 0.02 M ≈ 0.005 M) mirrored the BJH-derived most probable pore diameter sequence, where narrower mesopores restricted polyalkylated byproduct formation through spatial confinement effects. These structure-performance relationships aligned with earlier findings on acid site redistribution and pore architecture modulation.

## 5. Conclusions

The reduction in Si(1Al) correlated with decreased Brønsted acid site density, while the increase in Si(0Al) signified enhanced framework stability. This relationship is corroborated by pyridine-IR spectroscopy, which also reveals a “decline-recovery-decline” trend in Brønsted acidity, closely linked to the Si/Al compositional evolution.

The results of comprehensive characterization indicated that extra-framework Al species initially increase due to preferential removal of framework Al during early-stage modification, resulting in defect accumulation, reduced crystallinity, and decreased Brønsted acidity with concomitantly increased acid strength. As modification progressed, further elimination of extra-framework Al stabilized the framework, leading to improved crystallinity. However, excessive modifier concentration (>0.01 mol/L) results in accelerated dealumination exceeding silication rates, causing renewed structural destabilization, morphological disorder, and reduced crystallinity and acidity.

**Figure 13:**
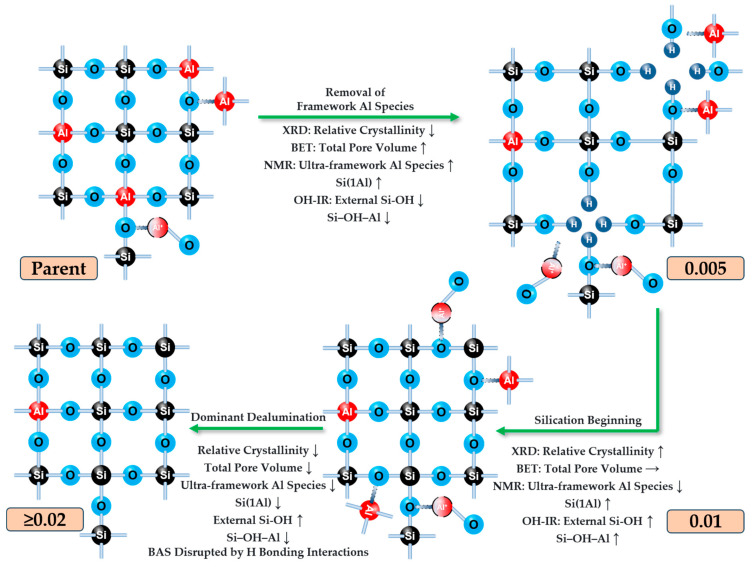
Hypothetical mechanism illustration of dealumination and silication of IM-5 zeolite treated with fluosilicic acid at different concentrations and its related characterization results.

This study demonstrates that hexafluorosilicic acid modification involves a multistep dynamic process: dealumination → defect formation → silication repair → Al redistribution. This mechanism underscores the self-healing capacity of aluminosilicate zeolites, achieving performance optimization through coordinated structural reconstruction and elemental rearrangement. These findings provide critical insights into the structure-property relationships of zeolite catalysts and guide the rational design of advanced modification strategies (Figure 13).

The optimal 0.01 M modifier achieves balanced trade-off between sufficient medium-strength acid sites and appropriate mesopore dimensions, which synergistically promote reactant accessibility while suppressing coking-induced deactivation.

The sustained activity of IM-5-0.01 correlated with its highest relative crystallinity (105.6% vs. 100% for parent), attributable to effective defect healing through silication as evidenced by ^29^Si MAS NMR.

## Figures and Tables

**Table 1:** The textural properties of the samples.

Samples	S_BET_^a^/ (m^2^·g^−1^)	S_micro_^b^/ (m^2^·g^−1^)	S_ext_^b^/ (m^2^·g^−1^)	V_total_^c^/ (cm^3^·g^−1^)	V_micro_^b^/ (cm^3^·g^−1^)	V_meso_^d^/ (cm^3^·g^−1^)
IM-5	372	332	40	0.32	0.15	0.17
IM-5-0.005	369	310	59	0.38	0.14	0.24
IM-5-0.01	365	307	58	0.38	0.14	0.24
IM-5-0.02	364	308	56	0.36	0.14	0.22
IM-5-0.03	359	303	56	0.37	0.14	0.23

a. BET Surface Area; b. t-Plot; c. Single point adsorption total pore volume of pores less than 92.5480 nm diameter at P/P_o_ = 0.98; d. V_total_ − V_micro_.

**Table 2:** Acid properties of the samples from NH_3_-TPD characterization.

Samples	n(SiO_2_)/n(Al_2_O_3_)		Acidity									
	Bulk ^a^	Framework ^b^	Total	Weak Sites			Medium Sites			Strong Sites		
				Amounts ^c^	Percents	T ^d^	Amounts ^c^	Percents	T ^d^	Amounts ^c^	Percents	T ^d^
IM-5	31	18	1463	629	43	188	278	19	270	556	38	403
IM-5-0.005	35	18	1366	628	46	198	342	25	328	396	29	428
IM-5-0.01	36	17	1249	587	47	197	475	38	363	187	15	431
IM-5-0.02	45	20	1036	487	47	195	186	18	314	363	35	412
IM-5-0.03	58	25	931	419	45	187	168	18	302	344	37	401

a. The bulk of SARs is determined by XRF; b. The framework of SARs is calculated by ^29^ Si MAS NMR; c. The unit is μmol/g; d. Desorption temperature.

**Table 3:** Acid properties of the samples from Py-IR characterization.

Samples	Brønsted Acid μmol/g			Lewis Acid μmol/g			B/L ^a^		
	190 °C	310 °C	410 °C	190 °C	310 °C	410 °C	190 °C	310 °C	410 °C
IM-5	206	180	132	49	47	36	4.2	3.8	3.6
IM-5-0.005	182	149	86	50	36	29	3.7	4.1	2.9
IM-5-0.01	206	176	100	50	45	35	4.1	3.9	2.8
IM-5-0.02	137	116	63	33	32	27	4.1	3.6	2.3
IM-5-0.03	118	102	50	23	25	23	5.2	4.1	2.2

a. the ratio of Brønsted Acid to Lewis Acid.

## Data Availability

The data presented in this study are available on request from the first or corresponding authors.

## Supplementary Materials

The following supporting information can be downloaded at: https://www.mdpi.com/article/10.3390/ma18102252/s1, Figure S1: The detailed schematic diagram of the NH_3_-TPD; Figure S2: The particle size distribution diagrams (length) of the samples; Figure S3: The particle size distribution diagrams (width) of the samples; Figure S4: NH_3_-TPD curves of the samples: (a) IM-5; (b) IM-5-0.005; (c) IM-5-0.01; (d) IM-5-0.02; (e) IM-5-0.03 and their deconvolution into Gaussian peaks. The black solid line is the experimental curve. The red dotted line is the cumulative curve of Gaussian peaks; Figure S5: The gas chromatographic elution curve of the pseudocumene alkylation reaction with methanol over the parent IM-5 zeolite when the time on stream (TOS) is 30 h.
