# Supplementary material for: Understanding the Effect of IM-5 Zeolite Treated with Hexafluorosilicic Acid for the Methanol Alkylation of Pseudocumene
Source: Materials (Basel). 2025 May 13;18(10):2252. doi: 10.3390/ma18102252 (PMC12113412; doi:10.3390/ma18102252)
Supplement: Supplementary file 1 [file materials-18-02252-s001.zip › materials-3575605-supplementary.pdf]

materials

Materials:

The organic structure directing agent 1,5 - bis(methylpyrrolidinium)pentane dibromide ($MPPBr_2$) (58.9 wt % purity) was obtained from Guangzhou Dayou Fine Chemical Factory. Sodium hydroxide (99 wt % purity), aluminum hydroxide (AR), fluorosilicic acid solution (40 wt% purity), ammonium chloride (99.5 wt % purity), 1,2,4 - trimethylbenzene (97 wt % purity), and methanol (99.5 wt % purity) were purchased from Beijing InnoChem Science and Technology Co., Ltd. Crude silica gel (with a solid content of 93.2 wt %) was from Qingdao Marine Chemical Factory. Deionized water, liquid nitrogen (99.999% V/V), helium (99.999% V/V), carrier gas nitrogen (99.999% V/V), combustion gas hydrogen (99.999% V/V), and combustion supporting gas compressed air were provided by Beijing Huanyu Jinghui Jingcheng Gas Technology Co., Ltd. The ammonia - helium mixture (10.012 % NH_3) was from Air Liquide (Tianjin) Co., Ltd. All materials were not further purified.

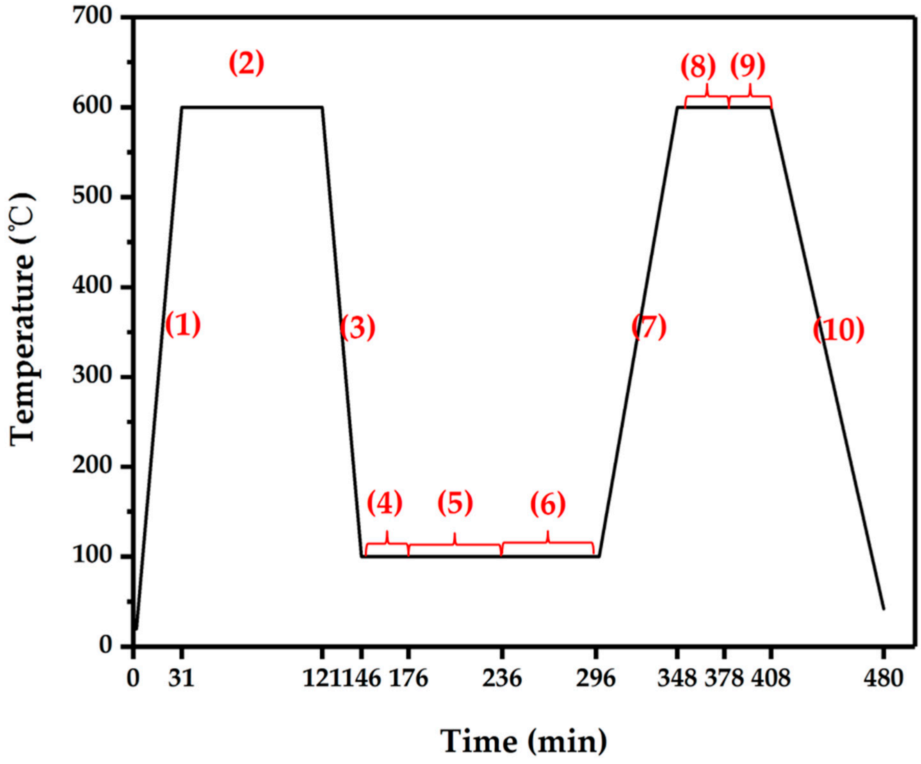

Figure S1. The detailed schematic diagram of the NH_3 - TPD. The serial numbers represent the following in sequence:

(1) Baseline adjustment for 2 minutes and heating from 20 °C to 600 °C at a rate of 20 °C per minute;

(2) Stabilization for 90 minutes;

(3) Cooling from 600 °C to 100 °C at a rate of 20 °C per minute. So the temperature at which NH_3 is adsorbed on the samples before subjecting them to the temperature ramp is 100 °C;

(4) Stabilization for 30 minutes;

(5) Changing the gas flow and waiting for 60 minutes;

(6) Changing the gas flow and waiting for 60 minutes;

(7) Baseline adjustment for 2 minutes and starting to record one data point every 1 second, heating from 100 °C to 600 °C at a rate of 10 °C per minute;

(8) Stabilization for 30 minutes;

(9) Stopping the recording and waiting for 30 minutes;

(10) Waiting for the temperature to drop to room temperature (about 42 °C).

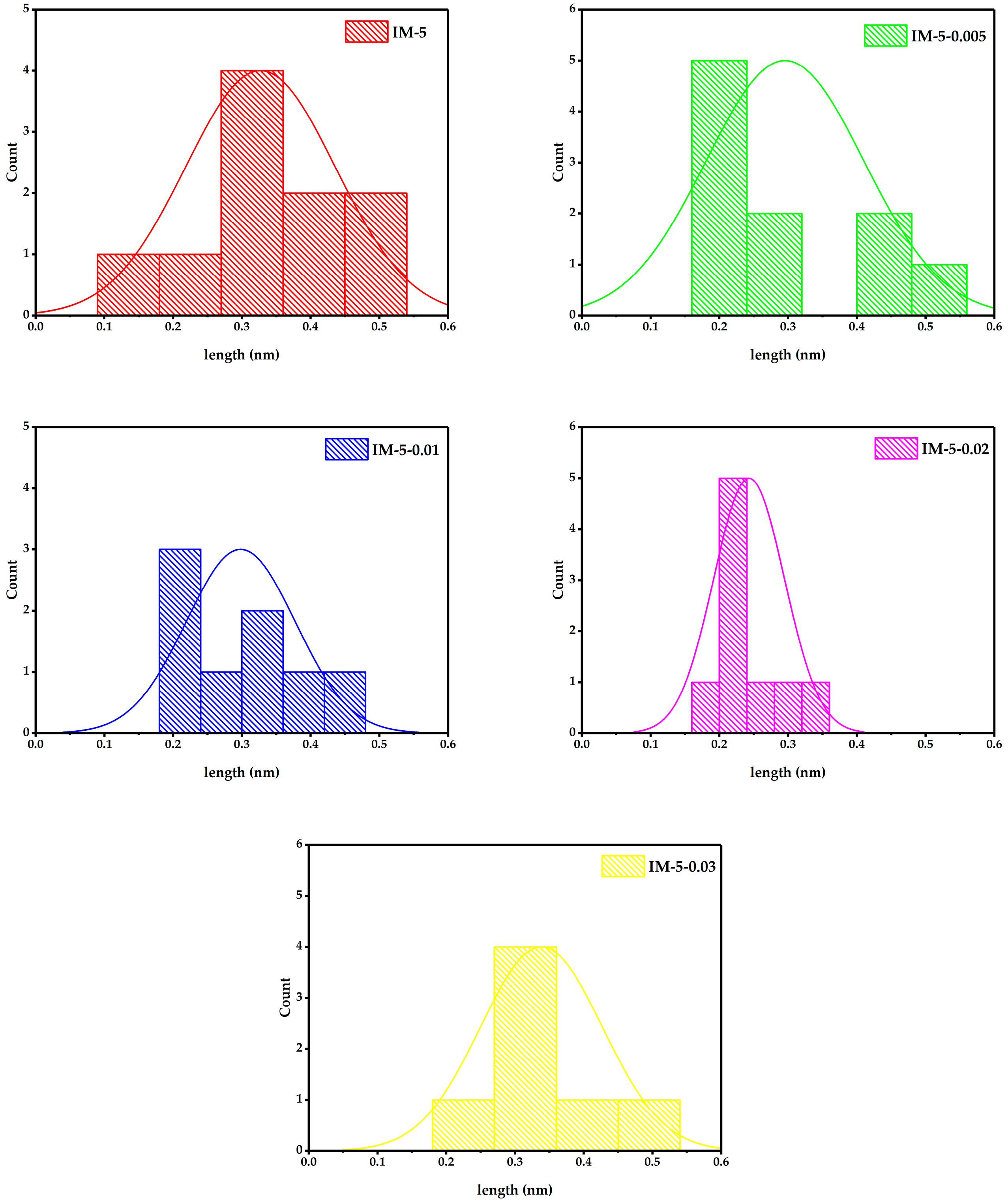

Figure S2. The particle size distribution diagrams (length) of the samples.

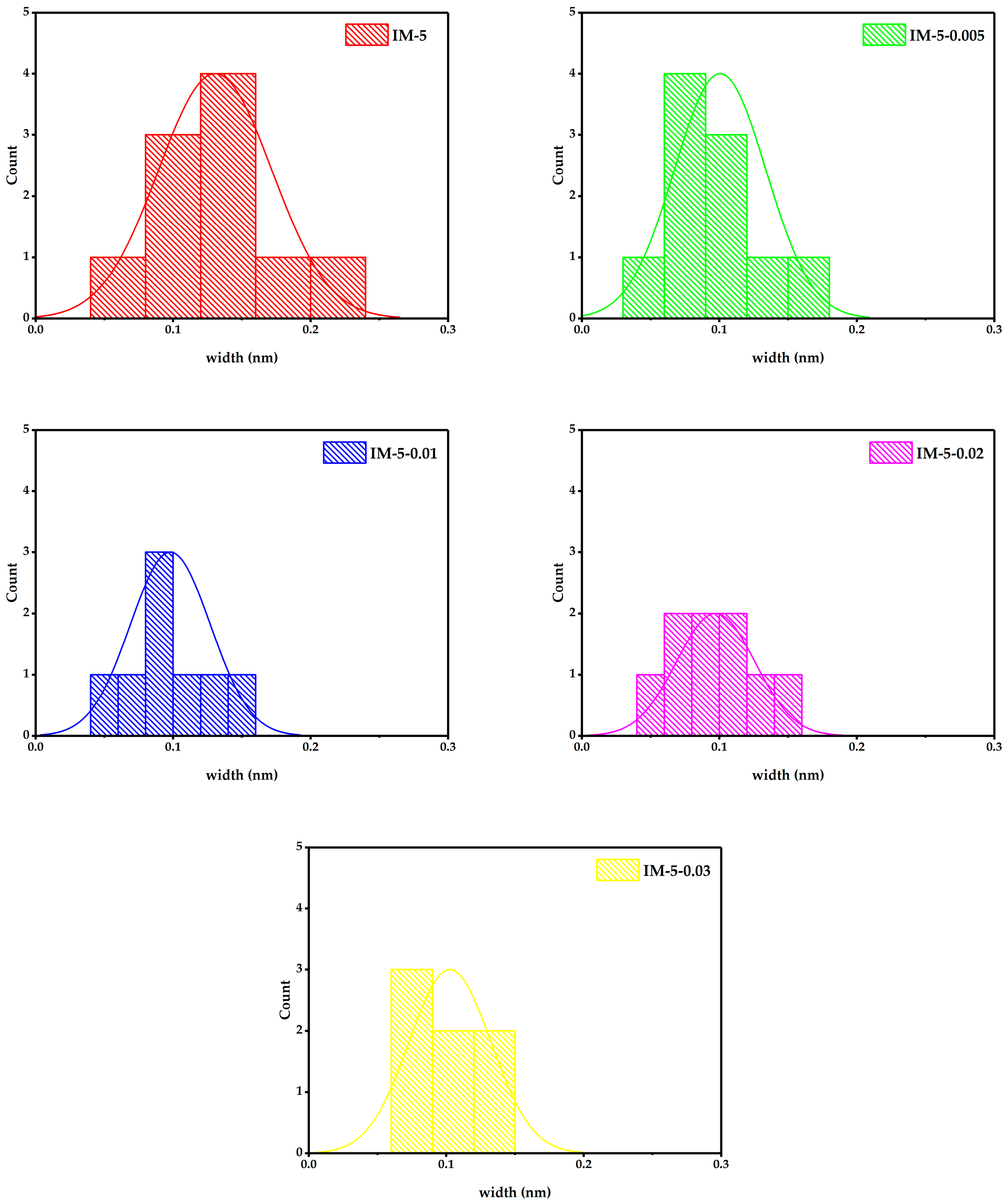

Figure S3. The particle size distribution diagrams (width) of the samples.

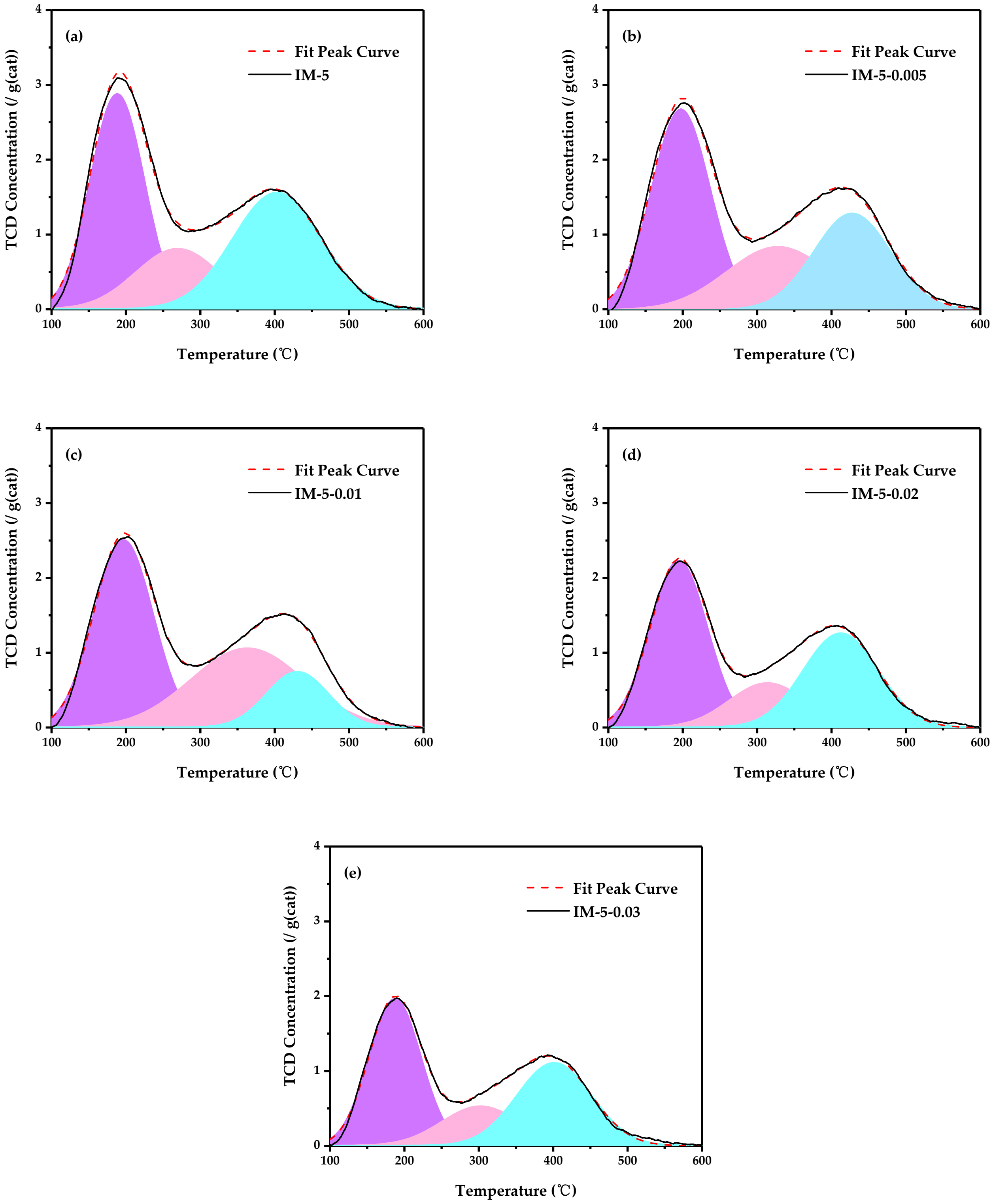

Figure S4. NH_3-TPD curves of the samples: (a) IM-5; (b) IM-5-0.005; (c) IM-5-0.01; (d) IM-5-0.02; (e) IM-5-0.03 and their deconvolution into Gaussian peaks. The black solid line is the experimental curve. The red dotted line is the cumulative curve of Gaussian peaks.

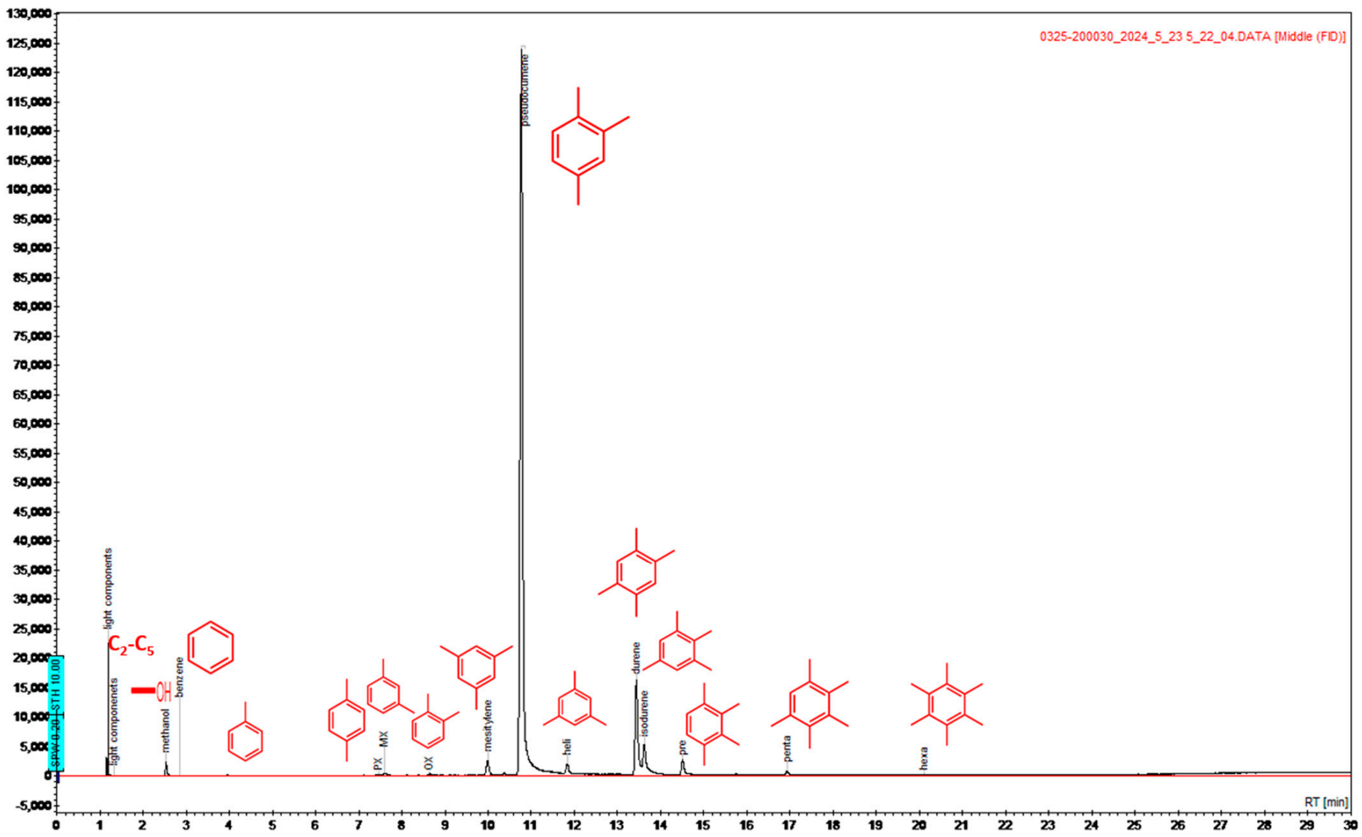

Figure S5. The gas chromatographic elution curve of the pseudocumene alkylation reaction with methanol over the parent IM-5 zeolite when the time on stream (TOS) is 30 hours.
